# Investigating the effectiveness of employing clinical faculty members in the shift work system during the Covid‐19 pandemic in Iran: A cross‐sectional study

**DOI:** 10.1002/hsr2.1010

**Published:** 2022-12-21

**Authors:** Abdolreza Gilavand

**Affiliations:** ^1^ Department of Education Development Center Ahvaz Jundishapur University of Medical Sciences Ahvaz Iran

**Keywords:** circadian, clinical faculty members, COVID‐19, Iran, shift work

## Abstract

**Introduction:**

Having an accurate attitude about shift work and choosing the right people to work in the shift work system increases organizational productivity and improves the employees' life quality. Consequently, the current research investigates the effectiveness of employing clinical faculty members in the shift work system during the Covid‐19 pandemic.

**Materials and Methods:**

In this descriptive study, 71 Iranian clinical faculty members with a history of shift work (night shift) during the Covid‐19 pandemic participated. Circadian Type Inventory (CTI) was used to collect data.

**Results:**

Based on the results of this research (Flexible/Rigid = Mean ± SD = 2.47 ± 18.17) and (Languid/Vigorous = Mean ± SD = 2.89 ± 15.35) which revealed that although clinical faculty members in normal conditions can overcome the feeling of sleepiness due to lack of sleep, they are not flexible and cannot work in the shift work system with the ability to stay awake at unusual times of the day or night. Pearson's correlation coefficient test also showed that as the value of the Languid/Vigorous component increases, the value of the Flexible/Rigid component also increases (*r* = 0.410). Likewise, there was no significant relationship between the main research variables and demographic characteristics.

**Discussion and Conclusion:**

Clinical faculty members who are simultaneously responsible for the duties of teaching students, research, and treating patients, if they are employed in a shift work system, especially during the pandemic, it may lead to a decrease in the quality of teaching, lack of motivation in research and indifference toward students' affairs, reducing flexibility, inability to update what they have learned in the professional world, increasing medical errors and also reducing their ability to manage the class. Consequently, it is suggested to avoid using them in the shift work systems as much as possible.

## 1 INTRODUCTION

Any work that can be done regularly and outside of the daily work period (7 a.m. to 6 p.m.) is called shift work.^1^ With the incident of the industrial revolution and the advancement of science and technology, particularly the invention of artificial light sources, human life changed, and a 24‐h society emerged. Due to economic, technological, social, and service needs, the organizations started working around the clock, 7 days a week.^1^, ^2^ A circadian rhythm is a physiological cycle that repeats approximately every 24 h. Many processes follow circadian rhythms, including the sleep‐wake cycle, hormonal changes, body temperature, and blood pressure.^3^

Shift work is one of the harmful factors of the work environment in the field of organizational ergonomics, it can have adverse effects on the productivity of the organization and the quality of human working life from various aspects.^4^ Shift work sleep disorder (SWSD), characterized by insomnia and excessive sleepiness associated with shift work, is one of shift workers' most common health problems. Shift work disorder causes insomnia, fatigue, worse work performance, increased risk of accidents and low quality of life. Furthermore, SWD is associated with reduced productivity and increased economic costs.^5^ Shift work has numerous harmful effects on employees' health, which can be mentioned such as the effect on the circadian and sleep‐wake cycles, adverse effects on family and social life, and long‐term effects, including digestive and cardiovascular problems and the risk of neuropsychiatric diseases.^6^ Shift workers, precisely women, are exposed to various mental problems, specifically depression symptoms.^7^, ^8^ The tendency to bad habits is more in jobs that are done at night. Shift work can change the pattern of food consumption. To fight fatigue and sleepiness, night workers tend to consume readily available food such as sausage or foods with high sugar, fat, and caffeine. Examining data and physiological mechanisms also indicates the relationship between shift work and the occurrence of gastrointestinal diseases. Shift workers give up their work activities due to physical and mental discomforts and turn to drug treatment to solve their problems. It has also been found that these people incline to smoke to deal with insomnia,^9^ and it has been revealed that disruption of the circadian rhythm is the cause of some cardiovascular risk factors such as hypertension and high blood fats.^10^

In Iran, the medical education system and the healthcare service delivery system have been merged, as a result of which there are currently 68 universities of medical sciences and healthcare services, whose clinical faculty members, in addition to teaching and research, are responsible for treating patients in teaching hospitals.^11^ Doctors who are obliged to work in shift systems are more exposed to wrong decisions in their career. They may have a more hostile attitude towards patients, make more medical mistakes, and have complicated relationships with their colleagues. Clinical faculty members who, as well as treating patients, are also responsible for teaching students and conducting research at the same time may have problems if they work in a shift work system. Consequently, having the right attitude about shift work and choosing the right people to work in the shift work system upsurges organizational productivity and improves the employees' life quality. Consequently, the current research investigates the effectiveness of employing clinical faculty members in the shift work system during the Covid‐19 pandemic

## 2 MATERIALS AND METHODS

This descriptive research was conducted for 6 months from October 23 to April 21, 2022, in teaching hospitals affiliated with Ahvaz Jundishapur University of Medical Sciences. Its statistical population included all clinical faculty members who had experienced the shift work system (Night shift from 8 p.m. to 8 a.m.) during the Covid‐19 pandemic. The sampling method was convenience sampling. Out of a total of 350 clinical faculty members of the university, 71 of them who were eligible to participate in the study participated in this research. Inclusion criteria also included being a clinical faculty member in one of the university's departments who had at least one year of service in shift work during the COVID‐19 pandemic. The Persian version of the Circadian Type Inventory (CTI) was used to collect data. This 11‐question questionnaire includes two independent factors: the first factor (including the first six questions) with the name (Flexible/Rigid) indicates the stability of the circadian rhythm. Those who score high on this factor are flexible and can work in the system shift work with the ability to stay awake at unusual times of the day or night. The second factor (including the second 5 questions) (Languid/Vigorous) indicates the range of circadian rhythm, a high score in the second factor includes weak people for whom it is difficult to overcome the feeling of sleepiness due to lack of sleep. For this purpose, people who score above 18.75 on the CTI questionnaire scale in the first factor, “Flexible/Rigid,” from 5 questions related to the FR factor are part of the 75th percentile. Therefore their circadian type is considered flexible in terms of rhythm. These people can stay awake at unusual times of the day and night. Also, people who score above 22.5 in the second factor called “Languid/Vigorous” out of 6 questions related to the LV factor are above the 75th percentile, and therefore their circadian type is considered weak in terms of range. It is more difficult for these people to overcome sleepiness. The validity and reliability of this questionnaire have been confirmed in Iran.^12^ Questionnaires were distributed electronically and through WhatsApp messenger among the clinical faculty members. The data were analyzed using SPSS version 24 software at a significance level of 0.05 and with the help of mean, standard deviation, percentage, independent *t*‐test, Kruskal−Wallis and analysis of variance.

## 3 RESULTS

Table 1 shows the characteristics of the demographic variables of the faculty members. Based on this table, 71 clinical faculty members participated in this research, 59.2% were men, and 40.8% were women. Likewise, regarding academic rank, 2.8% were instructors, 76.1% were assistant professors, 14.1% were associate professors, and 5.6% were professors.

**Table 1:** Demographic characteristics of faculty members

		Frequency	Percent
Gender	Man	42	59.2
	Woman	29	40.8
Academic rank	Instructor	2	2.8
	Assistant professor	54	76.1
	Associate professor	10	14.1
	Professor	4	5.6
Age	31−45	40	56.3
	46−55	24	33.8
	>55	7	9.9
Duration of employment as a faculty member	<5	23	32.4
	6−15	34	47.9
	16‐20	5	7.0
	21−30	9	12.7
Having a clinic = doctor's office	Yes	38	53.5
	No	33	46.5
Experience of getting Covid‐19	Yes	36	50.7
	No	35	49.3
Total		71	100.0

Table 2 shows descriptive indices of the leading research variables (questionnaire and its dimensions).

**Table 2:** Descriptive indices of the main research variables (questionnaire and its different dimensions)

Demographic characteristics		Flexible/Rigid	Languid/Vigorous	Total
Gender	Man	18.4 ± 2.61	15.71 ± 2.82	34.11 ± 4.6
	Woman	17.83 ± 2.24	14.83 ± 2.95	32.65 ± 4.29
	*p* Value	0.969 (0.336)	1.277 (0.206)	1.354 (0.18)
Academic Rank	Instructor	17.5 ± 0.71	16.5 ± 3.54	34 ± 4.24
	Assistant professor	18.39 ± 2.45	15.35 ± 3.08	33.74 ± 4.75
	Associate professor	18.1 ± 2.33	15 ± 2.26	33.1 ± 3.21
	Professor	17.25 ± 2.06	16 ± 2.16	33.25 ± 4.11
	*p* Value	1.02 (0.797)	0.842 (0.839)	0.106 (0.991)
Age	31−45	18.37 ± 2.48	15.32 ± 2.86	33.7 ± 4.61
	46−55	17.92 ± 2.52	15.12 ± 3.18	33.04 ± 4.7
	>55	17.86 ± 2.48	16.28 ± 2.06	34.14 ± 3.44
	*p* Value	0.388 (0.824)	1.746 (0.418)	1.094 (0.579)
Duration of employment as a faculty member	<5	18.96 ± 2.44	15.52 ± 2.91	34.48 ± 4.71
	6−15	17.85 ± 2.55	15.15 ± 2.78	33 ± 4.51
	16−20	18.2 ± 1.3	16 ± 5.15	34.2 ± 5.93
	21−30	17.33 ± 2.5	15.33 ± 2	32.67 ± 3.16
	*p* Value	2.908 (0.406)	0.194 (0.979)	2.057 (0.561)
Having a clinic = doctor's office	Yes	17.71 ± 2.23	15.87 ± 2.41	33.57 ± 3.88
	No	18.697 ± 2.65	14.76 ± 3.29	33.45 ± 5.19
	*p* Value	−1.703 (0.093)	1.635 (0.107)	0.115 (0.909)
Experience of getting Covid‐19	No	18.33 ± 2.65	15.39 ± 2.78	33.72 ± 4.71
	Yes	18 ± 2.29	15.31 ± 3.04	33.31 ± 4.33
	*p* Value	0.566 (0.573)	0.108 (0.914)	0.379 (0.706)
Total	‐	18.17 ± 2.47	15.35 ± 2.89	33.52 ± 4.5

The mean and standard deviation of the first factor was (Flexible/Rigid = Mean ± SD = 18.17 ± 2.47), which showed that during the Covid‐19 pandemic, clinical faculty members could not be flexible and work in a shift work system with the ability to stay awake at unusual times of the day or night.

The mean and standard deviation of the first factor was (Languid/Vigorous = Mean ± SD = 15.35 ± 2.89), which showed that during the Covid‐19 pandemic, clinical faculty members under normal conditions could overcome the feeling of sleepiness due to lack of sleep.

The relationship between the main variables of the research with demographic characteristics (such as gender, academic rank, age, duration of activity, and type of employment as a member of the academic staff and being infected with the Covid‐19 virus) was investigated using the independent *t*‐test and the Kruskal−Wallis test, which was not significant.

Table 3 examines the relationship between the dimensions of the CTI and the Pearson correlation coefficient test, revealing that with the increase in the value of the loose/lively component, the value of the flexible/inflexible component also increases (*r* = 0.410).

**Table 3:** Examining the relationship between the dimensions of the normal Circadian Type Inventory (CTI) each other

CTI		Flexible/Rigid	Languid/Vigorous
Flexible/Rigid	Pearson correlation coefficient	1	0.410
	*p* Value		<0.001
Languid/Vigorous	Pearson correlation coefficient	0.410	1
	*p* Value	<0.001	
Total	Pearson correlation coefficient	0.811	0.866
	*p* Value	<0.001	<0.001

## 4 DISCUSSION

LV and FR variables could act as predictive indices to select and employ individuals in a shift work system. The purpose of this research was to highlight the consequences of employing clinical faculty members in the shift work system during the Covid‐19 pandemic. The results of the investigation of the circadian rhythm revealed that although the faculty members in normal conditions can overcome the feeling of sleepiness and lack of sleep, they are not flexible and cannot work in the shift work system with the ability to stay awake at unusual times of the day or night. Studies have shown that shift work causes burnout and distress in personal life.^13^

Yadollahi et al. have investigated the major disorders and problems caused by shift work in nurses of teaching hospitals of Shahrekord University of Medical Sciences in the center of Iran and mentioned the disruption in personal, family, and social life as the most critical consequences. The current research also found that the forced employment of people in the shift work system without obtaining their consent causes a decrease in job satisfaction, which is consistent with our research.^14^ The results of the research of Farahnaki et al. among the nurses of the teaching hospitals of Ilam University of Medical Sciences in the west of Iran showed that the problems caused by shift work among the nurses had a high prevalence, which was mental problems, problems related to social life, and digestive problems, respectively. This research also found that people who voluntarily chose shift work have more job satisfaction than people who accepted work in the shift system by compulsion, which is consistent with our research.^15^ Hemmati‐Maslakpak et al.'s research on nurses working in teaching hospitals of Urmia University of Medical Sciences in northwest Iran also showed that job satisfaction and shift work have an inverse relationship.^16^

Chang et al. researched the effect of job satisfaction and sleep quality on shift nurses in teaching hospitals in Taiwan, which showed that job satisfaction and sleep quality were weaker in nurses who worked the night shift.^17^ The results of the research of Karagozoglu et al. in a medical center at İnönü University in Turkey showed that the sleep quality of shift nurses is low and their job satisfaction is average. As the quality of nurses' sleep decreases, their job satisfaction also decreases.^18^ The research of Josten et al. in the Netherlands showed that nurses who work 9‐h shifts have more fatigue and physical problems and are less satisfied with their working hours and free time.^19^ Sveinsdóttir's research revealed that Icelandic nurses who work in shifts are satisfied with their work and that their shift assignment did not pathologically disrupt their circadian cycle, which results inconsistent with our research.^20^ Rosa et al. conducted review research to investigate the effects of shift work and the non‐synchronicity of circadian rhythms on nurses' health. In this research, 24 articles were examined. They mentioned shift work as an obstacle to social and family relationships and a risk factor for stress, sleep disorders, metabolic disorders, diabetes, cardiovascular disorders, and breast cancer. They have also predicted that it may indirectly increase abortion, absenteeism, and stress caused by their work.^21^ Li et al. conducted a study to evaluate predictors of SWSD among nurses during the COVID‐19 pandemic in Chinese hospitals. They reported the prevalence of SWSD as 48.5%.^22^

### 4.1 Limitations and suggestions

Although a standard questionnaire was used in this study to measure the effectiveness of employing clinical faculty members in the shift work system during the Covid‐19 pandemic based and other methods are not suggested in it. However qualitative methods such as interviews can also be used to complete this tool. It is suggested to use these methods in future research.

## 5 CONCLUSION

Clinical faculty members who are simultaneously responsible for the duties of teaching students, research, and treating patients, if they are employed in a shift work system, especially during the pandemic, it may lead to a decrease in the quality of teaching, lack of motivation in research and indifference towards students' affairs, reducing flexibility, inability to update what they have learned in the professional world, increasing medical errors and also reducing their ability to manage the class. Consequently, it is clear that if they are assigned to the shift work system, it will result in job dissatisfaction and, consequently, a decrease in organizational productivity and quality of life. It is suggested to avoid using them in the shift work systems as much as possible.

## AUTHOR CONTRIBUTIONS

**Abdolreza Gilavand**: Conceptualization; data curation; formal analysis; funding acquisition; investigation; methodology; project administration; resources; software; supervision; validation; visualization; writing – original draft; writing – review and editing. All authors have read and approved the final version of the manuscript.

## CONFLICT OF INTEREST

The author declared no conflict of interest.

## ETHICS STATEMENT

This research was derived from a research project (Ethics Code of IR.AJUMS.REC.1400.404). Therefore, have been completely observed by the author.

## TRANSPARENCY STATEMENT

Abdolrea Gilavand affirms that this manuscript is an honest, accurate, and transparent account of the study being reported; that no important aspects of the study have been omitted; and that any discrepancies from the study as planned (and, if relevant, registered) have been explained.

## Data Availability

The original contributions presented in the study are included in the article/supplementary material, further inquiries can be directed to the corresponding author. The corresponding author had full access to all of the data in this study and takes complete responsibility for the integrity of the data and the accuracy of the data analysis.
